# Myxopapillary Ependymoma: A Case Report

**DOI:** 10.7759/cureus.84996

**Published:** 2025-05-28

**Authors:** Shervin Arjomand, James Park, Chul Chae

**Affiliations:** 1 Radiology, California University of Science and Medicine, Colton, USA; 2 Medical Imaging, Arrowhead Regional Medical Center, Colton, USA

**Keywords:** myxopapillary ependymoma, neuroradiology, primary spinal tumor, spinal cord tumor surgery, spinal tumor case report

## Abstract

Myxopapillary ependymoma (MPE) is a rare, slow-growing glial tumor classically arising from the filum terminale. Imaging studies play a crucial role in identifying this intradural mass, guiding the diagnostic and surgical approach. This case report details the clinical presentation, diagnosis, and treatment of a 48-year-old male presenting with lower back pain and urinary incontinence who was diagnosed with WHO grade II MPE. This study includes a comprehensive review of MPE, its imaging characteristics, histopathology, treatment options, and prognosis.

## Introduction

Ependymomas are tumors of the ependymal cells, which line the ventricles of the brain and central canal of the spinal cord, and are responsible for the production and regulation of CSF, which provides mechanical support for and cushions the entire central nervous system. Ependymomas are the most common intramedullary tumors, with previous studies estimating them to comprise 50-60% of intramedullary tumors [1,2]. Myxopapillary ependymoma (MPE) accounts for 13% of ependymomas, and predominantly affects the filum terminale, a thin thread-like structure at the end of the spinal cord (70-90% of cases), with a median age of 35 years and slight male predominance [3,4]. Traditionally classified as WHO grade I, MPE is now recognized to exhibit a broader biological spectrum, and as a result, has been upgraded to grade II to acknowledge its elevated proliferative activity and invasive features [5]. With an incidence of 1.00 per million person-years in the American population, MPE is a rare occurring tumor [6]. MPEs have similar molecular markers to other ependymomas, generally testing positive for GFAP, S100, and vimentin [5]. This case highlights the diagnostic presentation and therapeutic dilemmas in MPE.

## Case presentation

Patient history and presentation

A 48-year-old male with no significant past medical history presented to the emergency department (ED) for evaluation following abnormal findings on a recent lumbar MRI. The patient reported a two-year history of intermittent back pain, initially triggered while lifting cement as part of his construction work. More recently, three months prior to the initial presentation, he slipped and fell, which exacerbated his back pain. The pain improved prior to the initial presentation to the ED, as the patient described minimal back pain but noted persistent weakness in his legs. He also reported urinary symptoms, including urinary urgency and occasional urinary leakage, particularly when coughing, and fecal incontinence for the past three years. He denied radicular pain, saddle anesthesia, additional falls, paralysis, or sensory disturbances such as tingling or paresthesia. Physical examination revealed 5/5 motor strength in both upper and lower extremities, with deep tendon reflexes graded 2+ throughout. Sensory examination was notable for decreased sensation to pinprick on rectal exam but was otherwise unremarkable. A lumbar MRI was obtained by his primary care physician (PCP) due to concern for possible spinal cord or nerve root pathology. Following the MRI findings described and shown below, his PCP advised him to seek further evaluation at the hospital.

Initial imaging

**Figure 1:**
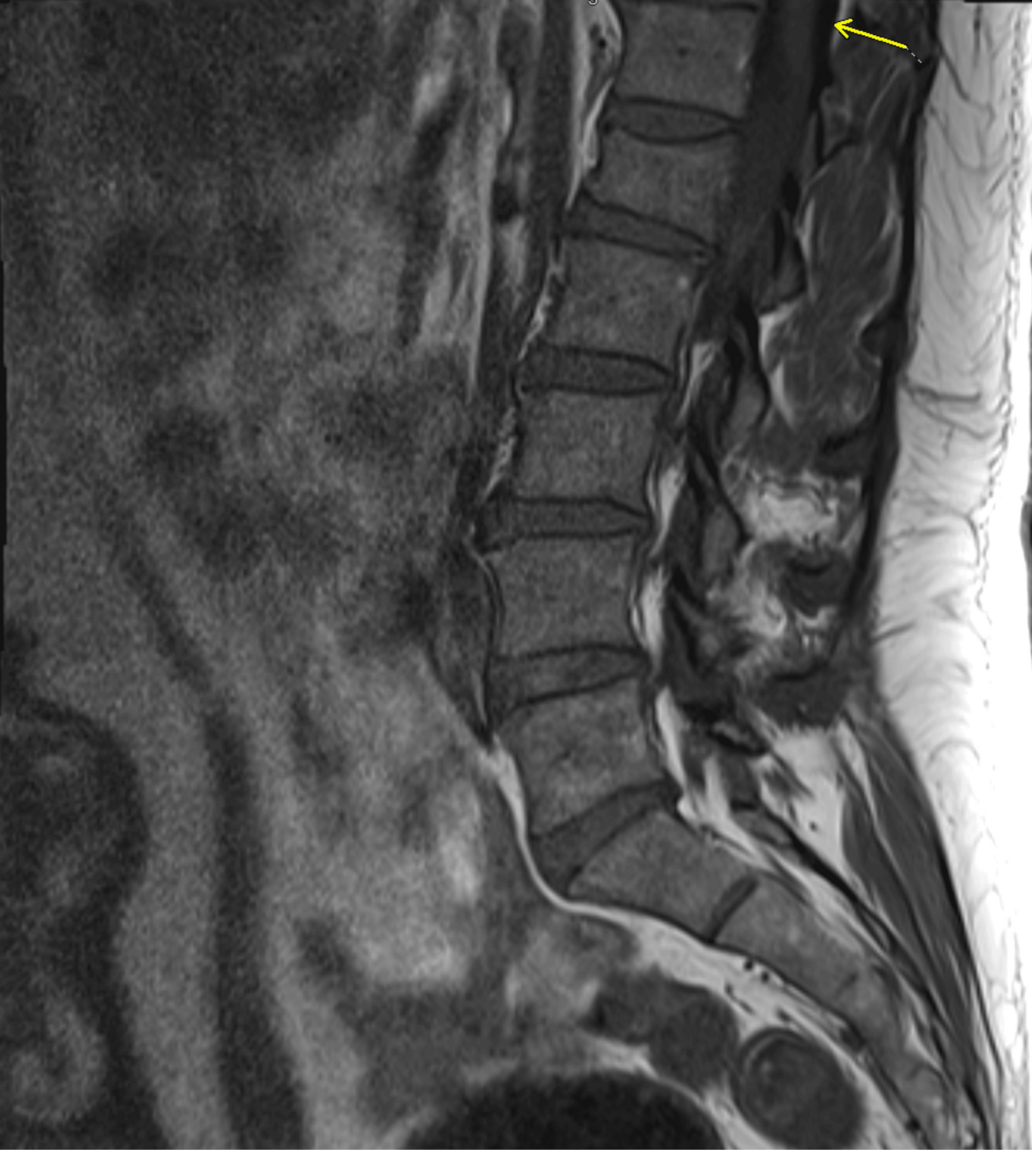
Sagittal T1 precontrast image

**Figure 2:**
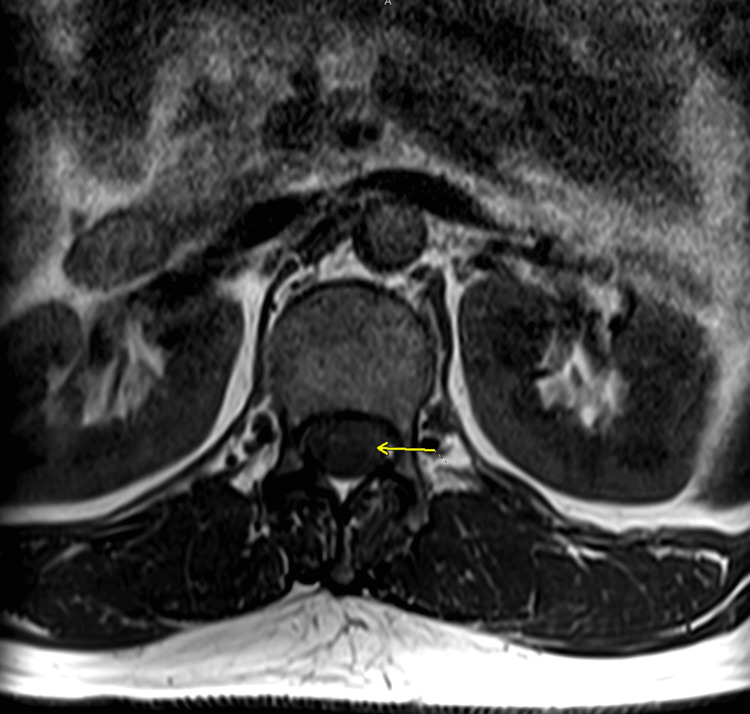
Axial T1 precontrast image at L2-L3

**Figure 3:**
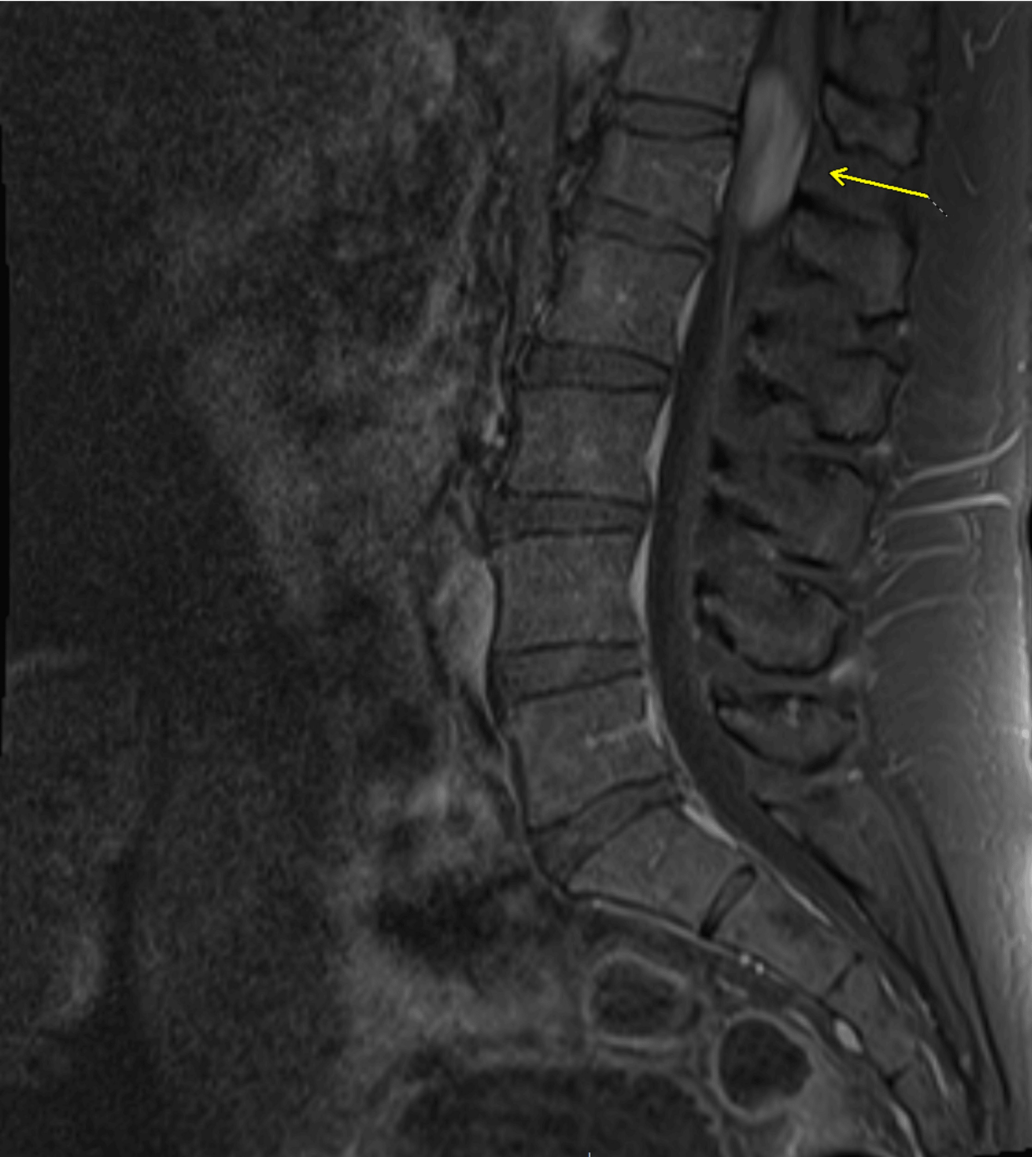
Sagittal T1 postcontrast image

**Figure 4:**
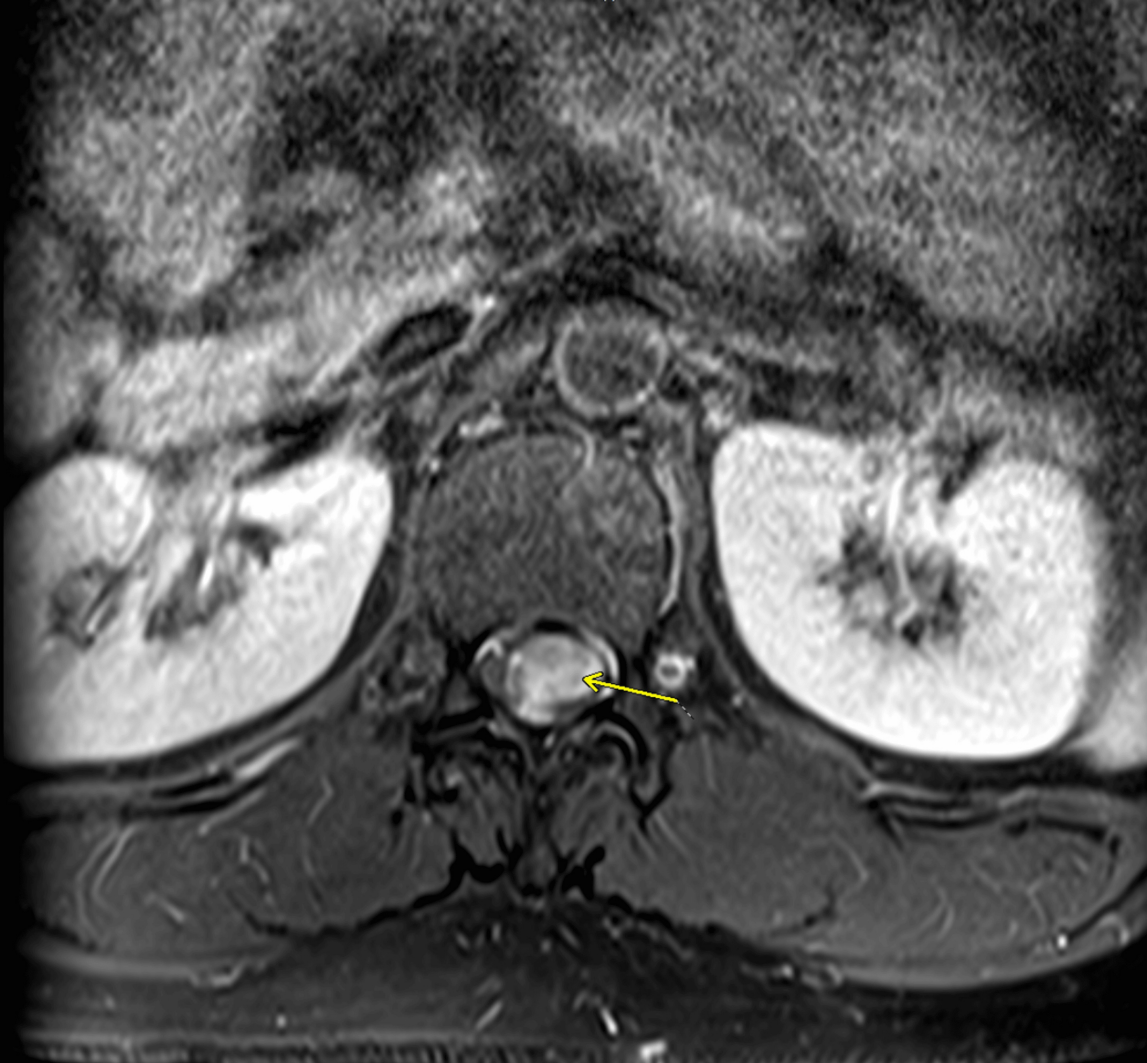
Axial T1 postcontrast image at L2-L3

**Figure 5:**
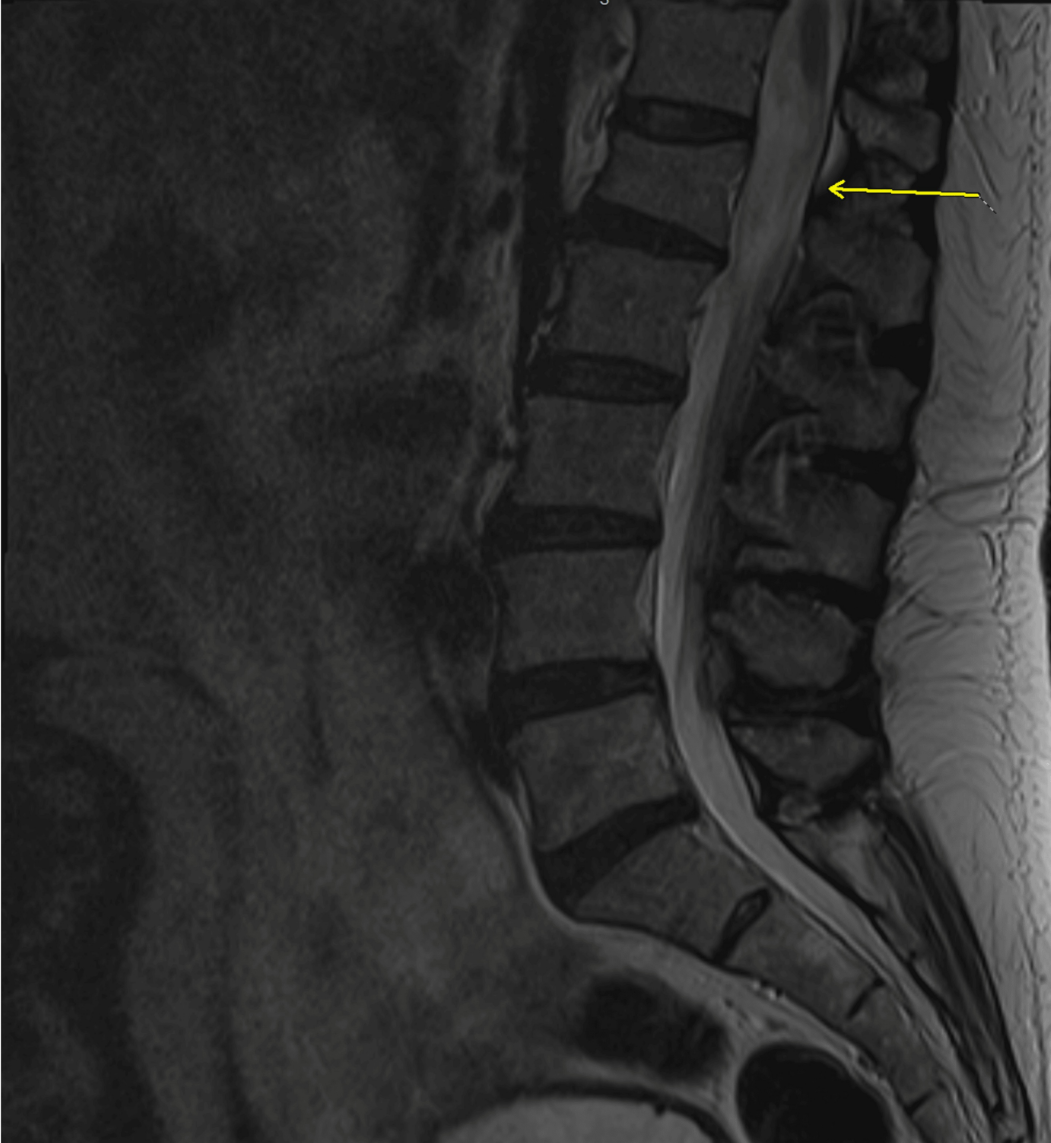
Sagittal T2 image

MRI of the thoracolumbar spine showed an enhancing mass from T12 to L1. MRI characteristics included homogenous enhancement consistent with myxopapillary ependymoma. These imaging findings align with the typical MPE presentation described in several studies on spinal ependymomas, which demonstrate isointensity to cord on T1-weighted images (Figures 1, 2) with homogenous postcontrast enhancement (Figures 3, 4), and hyperintensity to cord on T2-weighted (Figure 5) and short tau inversion recovery (STIR) images [2,3].

Hospital course

Upon initial neurosurgical consultation, the patient was informed of the need for surgical intervention to remove the mass. However, he opted to defer surgery and pursue close monitoring instead. After five months, the patient decided to move forward with surgery. He experienced no progression or worsening of his symptoms mentioned above.

Surgical intervention and pathology

The patient underwent partial laminectomy with tumor resection and gross total resection (GTR), defined as the removal of all visible tumor tissue, was achieved, although en bloc resection, which is a form of GTR in which the tumor is removed in a single, intact piece, was not possible due to the tumor's involvement with surrounding nerve roots [7,8]. Piecemeal GTR was done, in which the tumor capsule was violated to remove all segments of the tumor [7,8]. Surgical resection is the primary treatment for MPE, with GTR often resulting in a favorable prognosis versus subtotal resection [6,9,10]. The pathology report revealed an arrangement of cuboidal to elongated tumor cells around hyalinized fibrovascular cores in a papillary fashion, with the accumulation of myxoid material within microcysts, staining positive for GFAP (glial fibrillary acidic protein), and S100 protein. The cells exhibit large nuclei with irregular nuclear membranes and prominent nucleoli. This presentation is consistent with prior literature on MPE histology, which describes MPE as papillary elements arranged radially around hyalinized fibrovascular cores staining positive for the proteins mentioned above [4,5].

Postoperative course and management

The case was reviewed in a tumor board meeting with neurosurgery, medical oncology, and radiation oncology to assess the need for adjuvant treatment, given that the resection was not en bloc. Adjuvant radiation therapy was recommended; however, the patient declined, citing concerns of radiation causing other forms of cancer to occur. At the first postoperative visit, he reported stable urinary incontinence, decreased sensation in the right lower extremity, and mild drainage from the incision site. No bowel incontinence, weakness, fever, or chills were noted. Long-term follow-up with MRI and neurological assessments was recommended to monitor for recurrence due to the lack of adjuvant therapy.

## Discussion

This patient’s initial clinical presentation aligns with previous documentation, as Liu et al. reported in their study of 34 patients with MPE, where 82.4% reported back pain, 44.1% reported weakness, and 26.5% reported sphincter dysfunction, all of which our patient experienced [11]. For MPE, the greatest factor for long-term outcomes for these patients depends on the degree of surgical resection. A systematic review of 475 patients with MPE reveals a recurrence rate of 15.5% in those with GTR versus 32.6% in those who received subtotal resection, regardless of adjuvant therapy [9]. While current guidelines are clear on recommending adjuvant radiation therapy for patients with subtotal resection, they are not so clear on providing postoperative radiation therapy following GTR [12]. The same systematic review mentioned above reveals a minimal decrease in GTR recurrence with adjuvant radiation therapy versus without, showing a 15.6% versus 15.9% recurrence rate, respectively [9]. According to the study by Zhang et al., the 5-year progression-free survival (PFS) rate for patients who underwent GTR without receiving adjuvant radiation therapy was 82%, and the 10-year PFS rate was 77% [13]. Another study by Kotecha et al. reported a median relapse-free survival (RFS) of 17.2 years after GTR, with 5- and 10-year RFS rates of 72.3% and 54.0%, respectively [14]. Although there is minimal literature that describes differences in outcomes between piecemeal GTR vs. en bloc GTR in MPE, one article describing the long-term outcome of six cases of en bloc GTR and nine cases of piecemeal GTR revealed one incident of recurrence in piecemeal GTR versus zero incidences of recurrence of en bloc GTR [7,8]. These findings indicate that GTR alone may be adequate for long-term survival, although careful long-term follow-up is necessary due to the potential for late recurrences.

## Conclusions

This case describes a relatively common presentation of myxopapillary ependymoma. The patient’s chronic fecal and urinary incontinence, lower back pain, lower extremity weakness, and sensory exam findings all have been documented in prior literature as common symptoms of MPE. MRI imaging revealed an enhancing mass from T12-L1, which coincides with previously reported MPE findings to be a mass localized around the lumbosacral spinal cord and filum terminale. GTR of the spinal mass was achieved, which has been associated with positive long-term outcomes for patients with a relatively low risk of recurrence. This patient’s mass was not able to be removed en bloc, and very little research has been done to assess if there is a significant difference in outcomes between the en-bloc and piecemeal GTR, in part because MPE is such a rare diagnosis. Even though adjuvant radiation therapy was recommended, the limited literature available suggests that MPEs removed with GTR may not need adjuvant therapy. However, more research needs to be done to standardize treatment for this tumor.
